# Prevalence of Ocular Anomalies in Craniosynostosis: A Systematic Review and Meta-Analysis

**DOI:** 10.3390/jcm11041060

**Published:** 2022-02-18

**Authors:** Parinaz Rostamzad, Zehra F. Arslan, Irene M. J. Mathijssen, Maarten J. Koudstaal, Mieke M. Pleumeekers, Sarah L. Versnel, Sjoukje E. Loudon

**Affiliations:** 1 Department of Plastic and Reconstructive Surgery, Erasmus MC, University Medical Center, 3000 CA Rotterdam, The Netherlands; 2 Department of Ophthalmology, Erasmus MC, University Medical Center, 3000 CA Rotterdam, The Netherlands; 3 Department of Oral and Maxillofacial Surgery, Erasmus MC, University Medical Center, 3000 CA Rotterdam, The Netherlands

**Keywords:** ocular anomalies, orbital malformations, syndromic craniosynostosis, non-syndromic craniosynostosis, craniofacial disorders

## Abstract

Background: The aim of this study was to describe the ophthalmic abnormalities and their prevalence in craniosynostosis prior to craniofacial surgery. Methods: A systematic search was conducted on Medline OVID, Embase, Cochrane, Google Scholar, Web of Science Core Collection. Inclusion criteria were English papers, children aged <18 years with non-syndromic and syndromic craniosynostosis, case reports, case series, and case-control studies. A system of domains was established consisting of an anatomic and functional ophthalmic domain. A meta-analysis of single proportions was carried out using random effects model and pooled mean proportions with 95% confidence intervals (CI) were calculated. Results: Thirty-two papers analyzing 2027 patients were included. Strabismus was the most common anomaly in non-syndromic craniosynostosis: Horizontal strabismus was highest prevalent in unicoronal craniosynostosis (UCS) 19% (95% CI 9–32), followed by vertical strabismus 17% (95% CI 5–33). In syndromic craniosynostosis, horizontal strabismus was most prevalent in Crouzon syndrome 52% (95 CI 26–76), followed by Apert syndrome 50% (95% CI 42–58). Vertical strabismus was most prevalent in Saethre-Chotzen 60% followed by Muenke’s syndrome 36%. Furthermore, astigmatism was the second most reported outcome in non-syndromic craniosynostosis and highest prevalent in UCS 35% (95% CI 21–51). In syndromic craniosynostosis, astigmatism was most frequently seen in Crouzon syndrome 43% (95% CI 22–65), followed by Apert syndrome 34% (95% CI 14–58). Moreover, in syndromic craniosynostosis, 5–40% had a decrease in visual acuity (VA) ≤ 0.3 LogMAR in the better eye and 11–65% had a VA ≤ 0.3 LogMAR in at least one eye. Discussion: This review demonstrates the high prevalence of ocular anomalies in non-syndromic and syndromic craniosynostosis. A multidisciplinary and systematic approach is needed for the screening and optimal treatment of these conditions in a timely manner.

## 1. Introduction

Craniosynostosis is a congenital craniofacial disorder with a prevalence of 3.1–6.4 per 10,000 live births worldwide [1,2,3,4,5]. Craniosynostosis is defined as premature closure of one or more cranial sutures. It may occur as an isolated finding or as part of a syndrome [1,6]. Isolated craniosynostosis mainly involves a single suture, and includes, in descending order of frequency, sagittal, metopic, unicoronal and lambdoid craniosynostosis [6]. It can be classified as syndromic when craniosynostosis is in combination with the presence of additional clinical symptoms, such as Apert syndrome, Crouzon syndrome, Muenke syndrome and Saethre-Chotzen. Syndromic craniosynostosis account for 15–40% of the total cases of craniosynostosis [7]. In syndromic craniosynostosis, the coronal (unicoronal or bicoronal) sutures are often involved. In some cases, the genetic cause of the disease is not known (yet) and cannot be classified in non-syndromic or syndromic craniosynostosis, this is defined as complex (multisutural) craniosynostosis. The premature fusion restricts the normal growth of the skull, brain and face. It also affects the osseous structures in the peri-orbital zone leading to orbital malformations, such as hypertelorism, orbital dystopia or midface hypoplasia. In hypertelorism, interorbital distance is increased, resulting from enlargement of the ethmoid cells and bones [8,9], while it can also be caused by a meningo-(encephalocele) [5]. Orbital dystopia is any kind of abnormal displacement of the orbit and their contents. The displacement can occur in three different dimensional planes. In patients with vertical dystopia there is a discrepancy in the vertical position of the orbits which results in a malalignment of the eyes vertically. Furthermore, midface hypoplasia is a common feature in syndromic craniosynostosis, in which both the bones and soft tissues in the mid-portion of the face are underdeveloped in three dimensions [10,11]. These orbital malformations may have an effect on the position of the eyes and eyelids, which in turn can lead to ocular anomalies and eye motility disorders [10,11]. Ocular anomalies include proptosis, enophthalmos, exposure keratitis, lacrimal dysfunction, eyelid ptosis or refractive errors such as astigmatism [10,11]. Eye motility disorders include strabismus, which is one of the main causes of the development of amblyopia. Furthermore, the premature closure of the sutures can cause high intracranial pressure leading to optic disc swelling, e.g., papilledema. It is important that these ocular anomalies and eye motility disorders are detected and treated in a timely manner to prevent vision loss. From birth, the visual system develops and achieves its functional maturity at the age of six years, when binocular fusion is reached. A fully developed visual system is completed at the age of ten years [12,13]. For some eye conditions, the timing of treatment is crucial. For instance, the results of treatment of amblyopia are more successful when the child is under seven years old [14]. Although ophthalmic findings occur frequently in various craniofacial disorders, the accurate prevalence of ocular anomalies in this population is not known yet. Therefore, the objective of this review is to assess the prevalence of ocular anomalies in children with non-syndromic and syndromic craniosynostosis in a systematic approach.

## 2. Materials and Methods

This systematic review was carried out according to the preferred reporting items for systematic reviews and meta-analysis (PRISMA) statement [15]. Additionally, the performed systematic review was registered prospectively in the International prospective register of systematic reviews, PROSPERO with the following registration number: CRD42021249963.

### 2.1. Eligibility Criteria

Inclusion criteria: studies on humans, papers written in English, children with non-syndromic or syndromic craniosynostosis of which ophthalmic examinations were available prior to craniofacial surgery, children aged <18 years, descriptive studies such as case reports, case series and randomized controlled trials, furthermore cohort studies and case-control studies were included. No distinction was made in ethnicity or gender. Exclusion criteria: cross-sectional studies, systematic reviews, and meta-analysis. 

### 2.2. Information Sources and Search

A comprehensive search was performed by using MedLine Ovid, Embase, Web of Science Core Collection, Cochrane Central Register of Controlled Trials and Google Scholar. The databases were searched from their respective until December 2021. The full search is demonstrated in the Supplementary Material A. 

### 2.3. Study Selection

Firstly, two reviewers (P.R. and Z.A.) independently reviewed the title and abstract of all records to select all relevant studies. Secondly, full text of the selected studies were read and assessed independently by the two reviewers (P.R. and Z.A.) for meeting the eligibility criteria. Thirdly, P.R. and Z.A. both checked the reference list to see if there were additional relevant references. The program Endnote X9 was used for the references. 

### 2.4. Data Collection Process and Data Items

Data were extracted from the included studies. One author extracted relevant data from each study and another author independently checked all data. Data extraction included: general information about the paper, country, setting, year, participant characteristics, method of diagnosis of ocular anomalies. 

### 2.5. Classification of Syndromic versus Non-Syndromic Craniosynostosis

Non-syndromic craniosynostosis consisted of sagittal, metopic, unicoronal or lambdoid craniosynostosis. Multisutural craniosynostosis, without a known genetic diagnosis were also included in the non-syndromic group. Syndromic craniosynostosis constisted of Apert, Crouzon, Saethre-Chotzen, Pfeiffer syndrome, Carpenter, TCF12, craniofrontonasal syndrome and complex craniosynostosis with mutations in ERF-gene and IL11RA-gene. 

### 2.6. Classification of Ocular Anomalies and Orbital Malformations

In order to analyze the ocular anomalies, a system of domains was created. The ocular anomalies were categorized in two domains, namely (1) an anatomic and (2) a functional ophthalmic domain. Anatomic anomalies were defined as anatomical or adnexal anomalies that impair or are likely to impair the vision. Functional anomalies were defined as functional ocular anomalies that impair the vision.

1. Anatomic ophthalmic domain: neuro-ophthalmic (papilledema, optic disk anomalies), disorders of bony orbits, eyelid anomalies, other (e.g., lacrimal dysfunction, keratitis).
2. Functional ophthalmic domain: strabismus (horizontal and vertical), decrease in visual acuity, ptosis, amblyopia and refractive errors (anisometropia, hypermetropia, myopia and astigmatism).

### 2.7. Risk of Bias

The risk of bias was assessed using a JBI critical appraisal tool for case studies modified for this study. The following domains were assessed: inclusion criteria, validity of identification of the condition, reliability of the method of measuring, consecutive inclusion, reporting of demographics, reporting of clinical information, confounding factors, appropriate statistical analysis. Each of the above-mentioned items was assessed with yes, no, or unclear. If the study met the criteria, two points were given to that item, and it was defined as low risk of bias. If the study did not meet the criteria, or it was unclear, 0 or 1 points were given. The points for each item were added up, resulting in a total score. Studies with a total score of at least 17 points were rated as low risk of bias. A total score of twelve to seventeen points were rated as medium risk of bias, while studies which scored below 12 points were rated as high risk of bias. Studies were not excluded a priori based on quality reporting assessment.

### 2.8. Statistical Analysis

Descriptive statistics were used for the prevalences. Prevalences of ocular anomalies were extracted or calculated from the available data. The total number of ocular and orbital anomalies were divided by the total sample size of each specific disorder and were presented as (*n* = %). A meta-analysis of single proportions was carried out using the random effects model for the differrent ocular anomalies, and pooled mean proportions with 95% CI’s were calculated. A *p* value of <0.05 was defined as statistically significant. Data was converted using the Freeman-Tukey double arcsine transformation, to modify for the small sample sizes and possibly extreme proportions. Heterogeneity was evaluated by the I^2^ statistics [16]. The software program R version 4.1.2. for windows was used for the meta-analysis and forest plots. The GRADE certainty rating was used for evaluation of quality of evidence [17]. This consisted of risk of bias, inconsistency, indirectness, imprecision and publication bias [17].

## 3. Results

**Figure 1:**
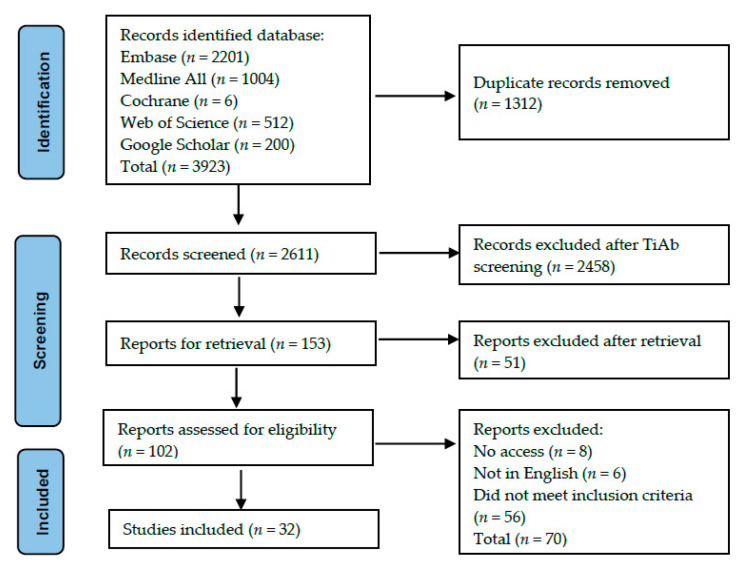
PRISMA diagram of the record selection process.

The literature search retrieved 3923 papers. After deduplication, 2611 papers were screened for eligibility. Of these, 2458 papers were excluded after ‘Title and Abstract’ (TiAb) screening. Hundred-two papers were available for full-text screening, of which eight papers had no access to full-text, six were not written in English and another 56 papers did not meet our inclusion criteria. In most of the excluded papers, ophthalmic examinations prior to craniofacial surgery were not described, or it was unclear if the patients had any craniofacial surgery prior to the ophthalmic examinations. Furthermore, other papers were excluded because the patients were older than 18 years old, or the outcome measurements were not based on ocular or orbital abnormalities. Thirty-two papers were eligible for inclusion and were included in the qualitative analysis. The detailed information of the record selection process is shown in Figure 1. Of the 32 papers included, 11 papers focused on non-syndromic craniosynostosis, 16 papers focused on syndromic craniosynostosis and five papers included both groups. The studies had no overlap in patients. The included studies were published between 1987 and 2021. The sample size ranged from 5 to 205 patients. A total of 2027 patients were included for analysis in this systematic review. In Table 1 the characteristics of the included studies are presented. In total 28 papers were included in the quantitive analysis. Four papers were excluded from the quantitative analysis, because they did not indicate the exact sample size per disorder. All meta-analysis and forest plots are demonstrated in the Supplementary Material B. 

### 3.1. Anatomic Ophthalmic Domain

#### 3.1.1. Non-Syndromic Craniosynostosis

All anatomical ophthalmic anomalies in non-syndromic craniosynostosis are presented in Table 2. Compared to syndromic craniosynostosis papilledema was less common in non-syndromic craniosynostosis, with a prevalence of 6% (*n* = 333; 95% CI 0–18) [18,19,20,21,22]. It had the highest prevalence in lambdoid craniosynostosis 67% (*n* = 9) reported by one study, which was also considered as an outlier in the analysis [19]. After exclusion of outliers, the pooled prevalence of papilledema in non-syndromic craniosynostosis was 1% (*n* = 324; 95% CI 0–5). Eyelid anomalies were only reported by two studies, in which epiblepharon was the most reported anomaly, with a prevalence of 50% in lambdoid craniosynostosis, 29% in sagittal and 26% in unicoronal craniosynostosis, respectively [23]. Lateral canthal dystopia had a prevalence of 14% in unicoronal craniosynostosis [24]. Among other anatomical ophthalmic anomalies, nasolacrimal duct obstruction was only reported in one study, showing a prevalence of 12% in unicoronal craniosynostosis [24]. There were two studies describing multisutural craniosynostosis (brachycephaly) without a known genetic diagnosis. The prevalence of papilledema in both studies was 50% (*n* = 2) [25] and (*n* = 4) [21].

#### 3.1.2. Syndromic Craniosynostosis

All anatomical ophthalmic anomalies in syndromic craniosynostosis are presented in Table 3. In syndromic craniosynostosis, papilledema was the most reported ophthalmic anomaly with a prevalence of 18% (*n* = 474; 95% CI 11–26) [18,20,21,25,26,27,28,29,30,31,32,33,34]. It was most prevalent in Crouzon syndrome, with a prevalence of 34% (*n* = 177; 95% CI 17–53) [20,21,25,26,27,29,30,34], followed by Apert syndrome, 9% (*n* = 59; 95% CI 1–22) [20,21,26,29,31,34]. One study showed a prevalence of 100% of papilledema in Apert syndrome [21]. However, this study only included one child with Apert syndrome and was considered as an outlier in the analysis [21]. After exclusion of outliers, the pooled prevalence of papilledema in Apert syndrome was 11% (*n* = 58; 95% CI 4–22). Anomalies of bony orbits, including proptosis, had a prevalence of 86% (*n* = 172; 95% CI 52–100) in syndromic craniosynostosis. It was most prevalent in Apert syndrome ranging between 87–100% [35,36], followed by Crouzon syndrome with a prevalence of 100% [37] and Pfeiffer syndrome 95% [33]. Furthermore, eyelid anomalies were reported in Saethre-Chotzen with a prevalence of 70% and in Muenke syndrome 67% [28].

### 3.2. Functional Ophthalmic Domain

#### 3.2.1. Non-Syndromic Craniosynostosis

All functional ophthalmic anomalies in non-syndromic craniosynostosis are presented in Table 4. Strabismus was the most reported anomaly in non-syndromic craniosynostosis. Horizontal strabismus was most prevalent in unicoronal craniosynostosis 19% (*n* = 199; 95% CI 9–32), with a prevelance of 10% (*n* = 120; 95% CI 2–21) of esotropia [23,38,39,40,41,42] and 11% (*n* = 192; 95% CI 5–19) of exotropia [23,38,40,41,42]. In the analysis of esotropia, the study of Vasco et al., 2008 was considered as an outlier [39]. After exclusion of outliers, the pooled prevalence of esotropia in unicoronal craniosynostosis was 8% (95% CI 3–6). Refractive errors were only reported by three studies. Astigmatism was highest prevalent in unicoronal craniosynostosis with a prevalence of 35% (*n* = 102; 95% CI 21–51) [23,38,40], followed by anisometropia 31% (*n* = 102; 95% CI 20–43) [23,38,40]. 

#### 3.2.2. Syndromic Craniosynostosis

All functional ophthalmic anomalies in syndromic craniosynostosis are presented in Table 5. Strabismus was the most reported anomaly consisting of horizontal (esotropia and exotropia) and vertical (hypotropia and hypertropia) strabismus with a prevalence of 58% (*n* = 445; 95% CI 41–73) [27,28,33,35,37,43,44,45,46]. Exotropia was most commonly reported in Crouzon syndrome with a prevalence of 47% (*n* = 197; 95% CI 18–77) [27,37,43,44,45], followed by Apert syndrome 38% (*n* = 128; 95% CI 27–50) [35,43,44,45]. Hypertropia was most prevalent in Saethre-Chotzen with a prevalence of 60%, followed by Muenke syndrome 36% [28]. Among refractive errors, astigmatism was the most reported outcome, and highest prevalent in Crouzon 43% (*n* = 136; 95% CI 22–65) [27,43,44,45], followed by Apert with a prevalence of 34% (*n* = 128; 95% CI 14–58) [35,43,44,45]. Ptosis was most prevalent in Saethre-Chotzen (90%), followed by Muenke syndrome (36%) [28]. Other ocular anomalies reported were nystagmus with a prevalence of 12% in Crouzon and the same study also reported blindness in 7% of the cases, whereas 46% of the other children had a poor vision in at least one eye [37]. 

### 3.3. Risk of Bias

**Figure 2:**
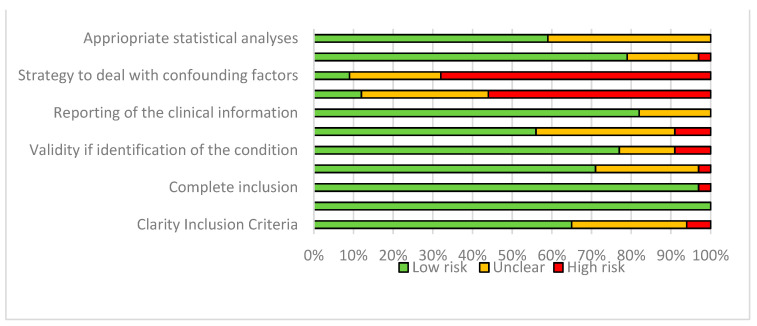
Risk of bias of included studies.

Figure 2 and Table 1. Present the assessment of risk of bias for the included studies. In total, six studies (19%) were evaluated as high risk of bias, ten studies (31%) were evaluated as medium risk of bias and 16 studies (50%) were assessed as low risk of bias. In most studies, a different method of ophthalmic examinations and diagnosis was used. Therefore, a high risk of confounding and performance bias was found in most studies. In total, 71% of the studies reported the method of ophthalmic examination and diagnosis. Therefore, the validity of diagnosis of the different anomalies was rated as low risk. The reliability of method of examination and diagnosis was evaluated as medium to high risk of bias in 26% of the studies. Demographics and clinical information were evaluated as low risk of bias, as most studies reported demographics such as age, gender, clinical situation at the moment of examination, medical history. Three studies did not define the demographics and were rated as high risk [24,36,37]. The included studies had a good representative population of the target population, most studies only evaluated syndromic or non-syndromic craniosynostosis, however some of these studies evaluated them together [18,20,21,25,47]. This often meant that the study population was larger, and therefore the prevalence’s were stated differently than if you compared each group with itself. Furthermore, not every study reported whether genetic analysis was performed to diagnose syndromic craniosynostosis, while this is officially necessary for establishing this diagnosis. Finally, for systematic reviews describing prevalence’s of rare diseases, it is difficult to take action on risk of bias. As sometimes it can be misleading to rate a study as low or high risk based on a previously compiled checklist, as some biases can be more valuable than others. Conclusively, the study by Hoy et al., 2012 indicates that the assessment of risk of bias provides invaluable information in the description of outcomes in systematic reviews of disease prevalence [48]. For these reasons, papers with high risk of bias were not excluded from the qualitative analysis. 

### 3.4. GRADE Certainty Rating

The meta-analysis included 23 separate analyses, which are demonstrated in the Supplementary Material B. Heterogeneity was considered low (<40%) in five analysis. This included horizontal strabismus in Apert syndrome I^2^ = 0%, *p* = 0,47, papilledema in Apert syndrome I^2^ = 11%, *p* = 0.35 and after exclusion of outliers I^2^ = 0%, *p* = 0.85, esotropia in UCS I^2^ = 16%, *p* = 0.31, and anisometropia in UCS I^2^ = 29%, *p* = 0.24. Heterogeneity was considered moderate (30–60%) in five analyses. This included astigmatism in UCS I^2^ = 54%, *p* = 0.11, papilledema in non-syndromic craniosynostosis I^2^ = 51%, *p* = 0.11, esotropia in UCS I^2^ = 50%, *p* = 0.09, exotropia in UCS I^2^ = 43%, *p* = 0.13, and exotropia in Apert syndrome I^2^ = 33%, *p* = 0.23. In the other 13 analysis, heterogeneity was considered high (65–100%) and statistically significant (*p* < 0.01). Conceivably caused by differences in patient characteristics or method of ophthalmic examination. Directness was considered high, as most studies directly investigated ocular anomalies in the target population. Imprecision cannot be ruled out due to the relatively small sample sizes and consequently wide confidence intervals. Furthermore, due to the small sample sizes and the rarity of craniosynostosis, it is difficult to draw a conclusion about publication bias. In addition, the meta-analysis was divided over the different ocular anomalies, so that the scatter plots cannot be officially used to indicate symmetry for publication bias. However, the study of Abboud et al., 2020 [19], was suspected of publication bias. As they reported a high prevalence of papilledema (67%) in lambdoid craniosynostosis, without further explanation. Finally, no exact conclusions can be drawn, as this grading is subjective, however, we rate the GRADE certainty rating as moderate.

## 4. Discussion

This systematic review is the first to report on the prevalence of pre-operative ocular anomalies in both non-syndromic and syndromic craniosynostosis in a systematically order. In order to systematically analyze the ocular anomalies, a system of domains was created by our workgroup. All ocular anomalies were subdivided into either an (1) anatomic ophthalmic domain or (2) a functional ophthalmic domain. Anatomic anomalies were defined as anatomical or adnexal anomalies that impair or are likely to impair the vision. Functional anomalies were defined as functional ocular anomalies that impair the vision. Current literature often used the ocular terms interchangeably throughout the papers and often there was no structure. This division has contributed to provide a more structured overview of the various ophthalmic abnormalities present in the patients. It must be noted that this is not an official classification and this classification was used because no previous classification system could be identified. Our study confirmed that ocular problems are highly prevalent in craniosynostosis. 

In the anatomic ophthalmic domain, papilledema was the most reported anomaly in both non-syndromic 1% (*n* = 333; 95% CI 0–18) [18,20,21,22] and syndromic craniosynostosis 18% (*n* = 474; 95% CI 11–26) [18,20,21,25,26,27,28,29,30,31,32,33,34]. In non-syndromic craniosynostosis, only one study reported papilledema in lambdoid craniosynostosis, with a prevalence of 67% (*n* = 9) [19]. This study was considered as an outlier and excluded from the quantitative analysis. In this study, two of the patients had minimal edema and four patients had low papilledema with no major vessel obstruction, without any further explanation of the reason of the high prevalence of papilledema in this group. In addition, it must be noted that there are several ways to diagnose papilledema. Namely by fundoscopy, Optical Coherence Tomography, and visual field examination. Therefore, assessment of papilledema can be subjective based on the accuracy and experience of the examiner. This also applies to other ocular anomalies.

Furthermore, in non-syndromic craniosynostosis, one study reported papilledema in unicoronal craniosynostosis with a prevalence of 3% [20]. In syndromic craniosynostosis, papilledema was most prevalent in Crouzon syndrome 34% (*n* = 177; 95% CI 17–53) [20,21,25,26,27,29,30,34] and Apert syndrome 11% (*n* = 58; 95% CI 4–22) [20,26,29,31,34]. There are several reasons for the high occurrence of papilledema in syndromic craniosynostosis. Papilledema is caused by high ICP as a result of premature closure of one or more sutures resulting in abnormal skull growth, ventriculomegaly, venous out-flow obstruction and obstructive sleep apnea [49]. Papilledema, can lead to visual loss if untreated [26,30]. Furthermore, epicanthal fold anomalies were reported in syndromic craniosynostosis in Saethre-Chotzen with a prevalence of 70% and in Muenke syndrome 67% [28]. Compared to a general population without craniofacial disorders, epicanthal fold is a common characteristic in Asian populations with a prevalence between 40–90%, while it is less often seen in non-Asian people with a prevalence of 2–5% [50]. 

In the functional ophthalmic domain, strabismus was the most prevalent ocular anomaly in both non-syndromic 24% (*n* = 443; 95% CI 12–38) [23,38,39,40,41,42,51,52] and syndromic craniosynostosis 58% (*n* = 445; 95% CI 41–73) [27,28,33,35,37,43,44,45,46]. Strabismus can occur primary, but also as a result of craniofacial surgery [23]. A reason for primary strabismus is shallow orbits, causing an increased risk of exocyclorotated orbits, which can lead to an incorrect insertion or even malformation of the extra-ocular muscles [23,53,54,55]. As malformation of the orbits, such as hypertelorism, vertical orbital dystopia and midface hypoplasia, are highly prevalent in syndromic craniosynostosis, this can be a reason of the high prevalence of primary strabismus in syndromic craniosynostosis. Our review confirmed that V-pattern exotropia was the most prevalent type of strabismus in syndromic craniosynostosis with a prevalence of 47% (*n* = 197; 95% CI 18–77) in Crouzon [27,37,43,44,45]. This is in line with the literature, as it has been stated that in Crouzon, a V-pattern exotropia is most prevalent due to extortion of the rectus muscle pulleys [56]. Whereas, esotropia was more common in non-syndromic craniosynostosis 8% (*n* = 113; 95% CI 3–16) in unicoronal craniosynostosis [23,38,39,40,41,42], in which usually the affected eye is ipsilateral to the fused suture [51]. Compared to the general population, these prevalence’s are much higher in patients with craniosynostosis. The global prevalence of strabismus in the general population was 1.93% (95% CI: 1.64–2.21), for exotropia it was 1.23% (95% CI: 1.00–1.46) and for esotropia 0.77% (95% CI: 0.59–0.95) respectively [57]. Subsequently, refractive errors were second highest prevalent in the functional ophthalmic group. Astigmatism and hypermetropia were the most common in both non-syndromic and syndromic craniosynostosis. Astigmatism can lead to amblyopia if untreated, in our review, amblyopia secondary to strabismus and refractive errors had a prevalence between 14–70% [28,33,35]. Amblyopia was not reported in non-syndromic craniosynostosis. This is not in line with previous studies, in which it has been stated that unicoronal craniosynostosis has an increased risk to develop amblyopia [58]. Correspondingly, the prevalence of amblyopia was higher in syndromic craniosynostosis compared to the general population. The global prevalence of amblyopia in the general Western population was 3.67% (95% CI: 2.89–4.45) [59].

Due to the rarity of craniosynostosis and the small numbers of patients in most studies, there are no clear guidelines in regard to age of screening, screenings technique and optimal time of ophthalmic treatment per disorder. There are currently few studies that have examined the ocular function pre- and postcraniofacial surgery. In a recent published prospective study of Ntoula et al., 2021 [38], 122 patients with non-syndromic craniosynostosis were examined pre- and postoperatively. They concluded that patients with sagittal craniosynostosis show a low prevalence of ocular anomalies, and therefore do not need to have a routine ophthalmic examination pre-operatively, and patients are advised to have ophthalmic examination postoperatively. However, they do advise patients with unicoronal and metopic craniosynostosis to have both pre- and postoperative ophthalmic examination, due to the high prevalence’s of ocular anomalies in these groups. Our review confirms the high prevalence of ocular anomalies in patients with unicoronal and lamdoid craniosynostosis. Moreover, in patients with metopic craniosynostosis we showed a high prevalence of astigmatism, which may lead to amblyopia left untreated. 

Furthermore, a recent retrospective study of Hinds et al., 2021 examined 165 patients with syndromic craniosynostosis, in which they analyzed the first and last ophthalmic examination [44]. In regard to the visual acuity, 76.7% of these patients had a best corrected visual acuity (BVCA) better than 0.3 LogMAR at their last examination, which can have a positive impact on the normal functioning of these patients. The study of Hinds et al., 2021 is a follow-up study of Khan et al., 2003, and both studies advise early screening and identification of ocular anomalies in syndromic craniosynostosis, irrespective if there are any ocular signs or complaints [43,44]. 

Systematic reviews describing a rare disease generally have limitations, which also applies to our study. The first limitation was the difference in sample size of the included studies, namely this ranged between 5–205, which results in different prevalence’s in regard to ophthalmic outcomes. In addition, the different types of craniosynostosis were often compared together, instead of individually, which makes it difficult to give an accurate prevalence of each ophthalmic outcome in each type of craniosynostosis. Furthermore, studies did not include healthy control groups to compare their study population with. Secondly, the method of ophthalmic examination and diagnosis, patient sample, in- and exclusion criteria were inconsistent in multiple studies. Assessment of ocular anomalies in children can be challenging, based on the accuracy and experience of the examiner. We expect that this might led to differences between the reported prevalence’s for each outcome. Thirdly, the focus of each study with regard to ophthalmic outcomes, was not the same between most papers, therefore, not all ophthalmic abnormalities were reported by every study. This led to missing data in the calculation of an average prevalence, and therefore, the calculated prevalence is not as accurate, as it would have been if more studies reported the same outcomes. For example, only one study reported dysfunction in lacrimal system in syndromic craniosynostosis, and only two papers reported this outcome for non-syndromic craniosynostosis, due to these small numbers, no generalized statement can be made for these ocular conditions. Finally, the GRADE certainty rating was assessed as moderate, due to the small number of studies and sample sizes and relatively high heterogeneity between the studies.

This systematic review shows the high occurrence of ocular anomalies in craniosynostosis. The aim of this review was to create awareness for the most prevalent ocular anomalies in a systematic order based on the two domains (1) anatomic ophthalmic domain and (2) functional ophthalmic domain. It is important to present this data on a well- organized and structured manner, so we can use this information to put more focus on finding a solution for optimal referral, screening, diagnosis, providing the required treatment and to develop new protocols. Based on the high occurrence of ocular anomalies in craniosynostosis as shown in our review, and based on the two recent studies on non-syndromic [38] and syndromic craniosynostosis [44], we can conclude that it is important to identify, screen and provide the necessary treatment to prevent any vision loss. However, no clear conclusion can yet be drawn at which age, each different type of disorders should be screened, by which technique. Future studies are needed, in which the ophthalmic conditions are examined in a prospective matter by an experienced orthoptist or ophthalmologist to prevent any performance bias. Furthermore, studies should include larger study samples, including multicenter studies (preferably internationally) to give more accurate data.

## Figures and Tables

**Table 1:** Study characteristics of the included studies.

Year	Author	Sample Size	Study Type	Bias Risk	Type of Craniosynostosis
2020	Abboud	9	RS	High	Lambdoid
2004	Albuquerque	5	CS	High	Apert
2008	Bannink	66	RS	Low	Apert, Crouzon, Pfeiffer
2015	Chung	88	RS	Low	Sagittal, lambdoid and unicoronal
1987	Diamond	34	RS	High	Unicoronal
2010	Florisson	205	RS	Low	Metopic, lambdoid, sagittal, unicoronal
1996	Gosain	14	PS	Medium	Unicoronal
2005	Gray	71	RS	Low	Crouzon
1993	Gupta	33	PS	Low	Apert, Crouzon, Metopic, sagittal, unicoronal, bicoronal
2021	Hinds	165	RS	Low	Apert, Crouzon, Pfeiffer, SC
2013	Imai	13	RS	High	Apert, Crouzon
2006	Jadico	21	RS	Low	Muenke, Saethre-Chotzen
2010	Jong, de	121	RS	Medium	Apert, Crouzon, Pfeiffer, Muenke, SC
2003	Khan	141	RS	Low	Apert, Crouzon, Pfeiffer, SC
2006	Khong	63	RS	Medium	Apert
2007	Khong	29	RS	Medium	Apert
2019	Kim	17	RS	Medium	Crouzon, Pfeiffer, Saethre-Chotzen
2010	Kreiborg	61	RS	High	Crouzon
2006	Liasis	8	CS	Medium	Apert, Crouzon, Pfeiffer
2007	MacIntosh	59	RS	Low	Unicoronal
1995	McCarthy	180	RS	Medium	Apert, Crouzon, CFS, Pfeiffer, metopic, sagittal, unicoronal
2021	Ntoula	122	RS	Low	Metopic, sagittal, unicoronal
2019	Ottenlander, den	38	PS	Medium	Muenke
2007	Ricci	38	PS	High	Metopic, sagittal, unicoronal
2015	Samra	79	RS	Low	Unicoronal
2016	Sharma	22	RS	Low	Pfeiffer
2016	Spruijt	37	RS	Low	Apert, Crouzon, Pfeiffer
1997	Stavrou	9	CS	Medium	Crouzon, metopic, sagittal, unicoronal, bicoronal
1996	Tuite	122	RS	Medium	Apert, Crouzon, Pfeiffer, SC, metopic, sagittal, unicoronal
2019	Van de Beeten	104	RS	Low	Muenke, Saethre-Chotzen, TCF12, CFS dysplasia, unicoronal
2008	Vasco	29	RS	Low	Metopic, sagittal, unicoronal
2020	Yu	24	RS	Low	Unicoronal

Abbreviations: RS = Retrospective case study; CS = case-series; PS = prospective study; CFS = Craniofrontonasal dysplasia.

**Table 2:** Anatomic ophthalmic anomalies in non-syndromic craniosynostosis.

Author	Sample Size	Synostosis	Neuro-Ophthalmic (%)	Eyelid (%)	Other
Abboud (2020)	9	Lambdoid	Papilledema 67	-	-
Chung (2015)	4	Lambdoid	-	Epiblepharon 50	-
	7	Sagittal	-	Epiblepharon 29 Lagophthal. ^1^ 14	-
	50	Sagittal + lamb. ^2^	-	Epiblepharon 26 Lagophthal. 8	-
	27	Unicoronal	-	Epiblepharon 26 Lagophthal. 7	-
Diamond (1987)	34	Unicoronal	Choroidal coloboma 3	Cant. Dystopia ^3^ 14	NLDO ^4^ 12 Microp.^5^ 3
Florisson (2010)	71 103	Metopic Sagittal	Papilledema 2 Papilledema 3	-	-
Gupta (1993)	3 4 7 7	All sutures Bicoronal Metopic Sagittal	Optic atrophy 67 Papilledema 50 Optic atrophy 14 Papilledema 14	-	-
Stavrou (1997)	2	Bicoronal	Papilledema 50	-	-
Tuite (1996)	10 29	Metopic Unicoronal	Papilledema 10 Papilledema 3	-	-
Van de Beeten (2019)	84	Unicoronal	Papilledema 0	-	-

Abbreviations: ^1^ lagophthalmus, ^2^ lambdoid synostosis, ^3^ lateral canthal dystopia, ^4^ nasolacrimal duct obstruction, ^5^ microphthalmus.

**Table 3:** Anatomic ophthalmic anomalies in syndromic craniosynostosis.

Author	Sample Size	Synostosis	Neuro-Ophthalmic (%)	Bony Orbits	Eyelid (%)	Other
Albuquerque (2004)	5	Apert	-	Proptosis 100	-	-
Bannink (2008)	66	Apert Crouz/Pf. ^1^	Papilledema 3 Papilledema 35	-	-	-
Gray (2005)	71	Crouzon	Papilledema 15 Optic atrophy 13	-	-	Cataract 2 Exp. K. ^2^ 15
Gupta (1993)	1 4	Apert Crouzon	Papilledema 100 Papilledema 25	-	-	-
Imai (2013)	8 5	Apert Crouzon	-	-	Entropion 50	Corn. E.^3^ 25 Corn. E. 40
Jadico (2006)	11 10	Muenke Sa-Ch. ^4^	Papilledema 9 Papilledema 20	Proptosis 18 Proptosis 10	Epicanth. ^5^ 67 Epicanth. 70	NLDO ^6^ 0 NLDO 60
Jong, de (2010)	22 45 28 26	Apert Crouz/Pf. Muenke Sa-Ch.	Papilledema 9 Papilledema 53 Papilledema 4 Papilledema 19	-	-	-
Khong (2006)	63	Apert	Optic atrophy 8 Coloboma disc 3	Proptosis 87	Entropion 2 Epibleph. ^7^ 3	Exp. K. 13
Khong (2007)	9 20	Apert Pro ^8^ Apert Ser ^9^	Optic atrophy 29 Optic atrophy 16	-	-	Kerato. ^10^ 21 Kerato. 25
Kim (2019)	15	Crouzon	Papilledema 13	-	-	-
Kreiborg (2010)	61	Crouzon	Optic atrophy 22	Proptosis 100	-	Exp. K. 12 Exp. Co. ^11^ 52
Liasis (2006)	8	Apert Pfeiffer	Papilledema 13 ODA 13	-	-	Exp. K. 0
Ottenlander (2019)	38	Muenke	Papilledema 9	-	-	-
Sharma (2016)	22	Pfeiffer	Papilledema 5	Proptosis 95	Entropion 5	-
Spruijt (2016)	18 19	Apert Crouz/Pf.	Papilledema 11 Papilledema 58	-	-	-
Stavrou (1997)	4	Crouzon	Papilledema 50	-	-	-
Tuite (1996)	10 19 4 22	Apert Crouzon Pfeiffer Sa-Ch.	Papilledema 20 Papilledema 37 Papilledema 25 Papilledema 5	-	-	-
Van de Beeten (2019)	7 4	Muenke Sa-Ch.	Papilledema 0 Papilledema 0	-	-	-

Abbreviations: ^1^ Crouzon & Pfeiffer, ^2^ exposure keratitis, ^3^ corneal erosion, ^4^ Saethre-Chotzen, ^5^ epichanthal anomaly, ^6^ nasolacrimal ductobstruction, ^7^ epiblepharon, ^8^ Pro-253-Arg type, ^9^ Ser252-Trp type, ^10^ keratopathy, ^11^ exposure conjunctivitis.

**Table 4:** Functional ophthalmic anomalies in non-syndromic craniosynostosis.

Author	Sample Size	Synostosis	Strabismus (%)		VA (LogMAR)	Ptosis (%)	Refractive Errors (%)
			Horizontal	Vertical			
Chung (2015)	4	Lambdoid	Esotropia 0 Exotropia 14	Hyper/Hypo ^4^ 0	-	-	Astigm ^1^. 0 Aniso. ^2^ 0
	7	Sagittal	Esotropia 0 Exotropia 14	Hyper/Hypo 14	-	-	Astigm. 29 Aniso^.^ 0 Hyperm ^3^.14
	50	Sagittal + lambdoid	Esotropia 14 Exotropia 30	Hyper/Hypo. 4	-	-	Astigm. 32 Aniso. 22 Hyperm.28 Myopia 8
	27	Unicoronal	Esotropia 19 Exotropia 22	Hyper/Hypo. 4	-	-	Astigm. 48 Aniso. 26 Hyperm.33
Gosain (1996)	14	Unicoronal	-	Hyper/Hypo. 57	-	-	-
Mac- intosh (2007)	59	Unicoronal	Esotropia 8 Exotropia 7	Hyper/Hypo. 7	-	-	Astigm. 25 Aniso. 39
Ntoula (2021)	22	Metopic	Eso/exo ^5^ 0	-	-	-	Astig. R. ^6^ 45 Astig. L. ^7^ 35 Aniso.^8^ 5
	84	Sagittal	Exotropia 4	-	-	-	Astig. R. 40 Astig. L. 46 Aniso. 1
	16	Unicoronal	Esotropia 6 Exotropia 19	-	-	-	Astig. R. 36 Astig. L. 43 Aniso. 21
Ricci (2007)	12	Metopic	Eso/exo 0	Hyper/Hypo 18	Abnormal in 8	-	-
	15	Sagittal	Eso/exo 0	-	Abnormal in 20	7	-
	11	Unicoronal	Eso/exo 0	-	Abnormal in 27	-	-
Samra (2015)	79	Unicoronal	Exotropia 14	Hyper.16	-	-	-
Vasco (2008)	12 7	Sagittal Unicoronal	- Esotropia 43	Hyper.29	-	8 -	- -
Yu (2020)	24	Unicoronal	Strabismus undefined 21	-	-	-	-

Abbreviations: ^1^ astigmatism, ^2^ anisometropia, ^3^ hypermetropia, ^4^ Hyper/hypotropia, ^5^ eso/exo: no distinguishment between esotropia and exotropia, ^6^ R = right eye, ^7^ L = left eye, ^8^anisometropia.

**Table 5:** Functional ophthalmic anomalies in syndromic craniosynostosis.

Author	Sample Size	Synostosis	Strabismus		VA (LogMAR)	Ptosis	Amblyopia	Refractive Errors
			Horizontal	Vertical				
Gray (2005)	71	Crouzon	Esotropia 8 Exotropia 23 Ex.+ hyper 3 Ex.+ hypo 3	Hyper. 1 Hypo. 1	32% ≤ 0.3 in at least one eye	-	21	Astigm. 59 Hyperm.59 Myopia 18
Hinds (2021)	57	Apert	Esotropia 3 Exotropia 47	-	-	-	-	Astigm. 54
	60	Crouzon	Esotropia 7 Exotropia 35	-	-	-	-	Astigm. 35
	14	Pfeiffer	Esotropia 0 Exotropia 36	-	-	-	-	Astigm. 36
	34	Saethre-Chotzen	Esotropia 18 Exotropia 21	-	-	-	-	Astigm. 38
Imai (2013)	8 5	Apert Crouzon	Exotropia 25 Exotropia 60	-	-	-	-	Astigm. 13 Astigm. 20
Jadico (2006)	11	Muenke	Eso/exo 55	Hyper. 36	-	36	18	Astigm. 9 Hyperm. 27 Myopia 18
	10	Saethre-Chotzen	Eso/exo 70	Hyper 60	-	90	70	Astigm. 50 Hyperm. 40 Myopia 30
Khan (2003)	141 ^1^	Apert	Esotropia 49 Exotropia 34	-	65% of total pt ≤ 0.3 one eye. 40%≤ 0.3 of total pt in better eye.	-	-	Astigm. 52
		Crouzon	Esotropia 20 Exotropia 47					Astigm. 43
		Pfeiffer	Esotropia 16 Exotropia 79					Astigm. 45
		Saethre-Chotzen	Esotropia 29 Exotropia 24					Astigm. 43
Khong (2006)	63	Apert	Esotropia 17 Exophoria 3 Exotropia 33	Hyper/ Hypo 5	5% ≤ 0.3 in better eye. 11% ≤0.3 one eye.	22	14	Astigm. 29 Aniso. 19 Hyperm. 24 Myopia 10
Khong (2007)	9	Apert *Pro253-Arg type*	Strabismus undefined 39	-	13 ≤ 0.3 one eye	-	20	-
	20	*Ser252-Trp type*	Strabismus undefined 47	-	60% ≤ 0.3 one eye.	-	56	-
Kreib- org (2010)	61	Crouzon	Exotropia 77	-	46% ≤ 0.3 one eye	-	-	-
Sharma (2016)	22	Pfeiffer	Esotropia 14 Exotropia 32	-	14% ≥ 0.3 ≤ 0.5 one eye	-	14	Astigm. 18 Hyperm.27 Myopia 9

Abbreviations: ^1^ No distinguishment was made between number of patients in all groups. Abbreviations: ex. = exotropia; hyper = hypertropia; hypo = hypotropia; astigm = astigmatism; hyperm. = hypermetropia, aniso=anisometropia.

## Data Availability

Data from the systematic review and meta-analysis is included in the paper or Supplementary Materials A and B.

## Supplementary Materials

The following supporting information can be downloaded at: https://www.mdpi.com/article/10.3390/jcm11041060/s1. Supplementary file A: Electronic database search. Supplementary file B. Forest plots and meta-analysis.
