# Supplementary material for: Prevalence of Ocular Anomalies in Craniosynostosis: A Systematic Review and Meta-Analysis
Source: J Clin Med. 2022 Feb 18;11(4):1060. doi: 10.3390/jcm11041060 (PMC8877705; doi:10.3390/jcm11041060)

Supplementary file A. Electronic database search

Embase

('eye disease'/exp OR 'hypertelorism'/de OR 'heterotopia'/de OR 'midface hypoplasia'/de OR 'visual system function'/exp OR (vision OR visual OR ptosis OR asthenopia OR eyestrain OR conjunctivit* OR keratitis OR keratoconus OR aniridia OR Anophthalmos OR Blepharophimosis OR Choroidal-Effusion* OR Coloboma OR Ectopia-Lentis OR Familial-Exudative-Vitreoretinopathies OR Hydrophthalmos OR Microphthalmos OR Persistent-Hyperplastic-Primary-Vitreous OR retinitis OR retinoblastoma OR Choroideremia OR Coloboma OR Vitreoretinopath* OR Opthalmopath* OR Leber-Congenital-Amaurosis OR hyphema OR endophthalmitis OR scleritis OR albinism OR ((refract*) NEAR/3 (error*)) OR ((drooping OR ptotic*) NEAR/3 (eyelid*)) OR blepharoptosis OR blepharo-ptosis OR ((midface*) NEAR/3 (hypoplas* OR retract*)) OR heterotopia* OR dystopia* OR hyperteloris* OR opthalmopath* OR oculopath* OR glaucom* OR ((eye* OR ocular* OR opthalm* OR lens OR lacrimal* OR strabismus* OR optic* OR pupil* OR retina OR retinal OR sclera OR scleral OR iris OR uveal OR uvea OR visual* OR orbit* OR conjunctiv* OR accomodat* OR cogan* OR corneal OR fraser OR aicardi OR cone OR Duane-Retraction OR gyrate OR Stargardt OR Walker-Warburg OR Weill-Marchesani OR choroid* OR vitreous) NEAR/3 (finding* OR diseas* OR malform* OR disorder* OR abnorm* OR disturb* OR neovascular* OR infect* OR inflammat* OR injur* OR irritat* OR red OR redness OR dry OR movement* OR pain OR swell* OR toxic* OR hemorrhage* OR fibros* OR prurit* OR tumor* OR tumour* OR myositis OR edema OR syndrom* OR discomfort* OR discharge* OR jaundic* OR fatigue* OR neoplas* OR cancer* OR leukoma OR dystroph* OR cell-loss OR neovasculariz* OR opacit* OR dysplas* OR dystroph* OR atroph* OR hypoplas* OR degenerat* OR ulcer* OR hypertension* OR hypotension*))):ab,ti,kw) AND ('craniofacial synostosis'/de OR 'plagiocephaly'/de OR 'brachycephaly'/de OR (saethre-chotzen* OR TCF12 OR heterotop*-ossificat* OR ((apert OR chotzen OR pfeiffer OR craniofrontonas* OR crouzon OR muenke OR ERF OR Carpenter* OR IL11RA) NEAR/3 (syndrom* OR diseas*)) OR trigonocephal* OR plagiocephal* OR brachycephal* OR craniostenos* OR craniosynostos* OR ((cranial* OR cranio* OR face* OR facial*) NEAR/3 (synostos*))):ab,ti,kw) AND (child/exp OR adolescent/exp OR adolescence/exp OR 'child behavior'/de OR 'child parent relation'/de OR pediatrics/exp OR childhood/exp OR 'child nutrition'/de OR 'infant nutrition'/exp OR 'child welfare'/de OR 'child abuse'/de OR 'child advocacy'/de OR 'child development'/de OR 'child growth'/de OR 'child health'/de OR 'child health care'/exp OR 'child care'/exp OR 'childhood disease'/exp OR 'child death'/de OR 'child psychiatry'/de OR 'child psychology'/de OR 'pediatric ward'/de OR 'pediatric hospital'/de OR 'pediatric anesthesia'/de OR 'pediatric intensive care unit'/de OR 'neonatal intensive care unit'/de OR 'prematurity'/de OR (adolescen* OR preadolescen* OR infan*

Medline

(exp Eye Diseases/ OR Hypertelorism/ OR (vision OR visual OR ptosis OR asthenopia OR eyestrain OR conjunctivit* OR keratitis OR keratoconus OR aniridia OR Anophthalmos OR Blepharophimosis OR Choroidal-Effusion* OR Coloboma OR Ectopia-Lentis OR Familial-Exudative-Vitreoretinopathies OR Hydrophthalmos OR Microphthalmos OR Persistent-Hyperplastic-Primary-Vitreous OR retinitis OR retinoblastoma OR Choroideremia OR Coloboma OR Vitreoretinopath* OR Opthalmopath* OR Leber-Congenital-Amaurosis OR hyphema OR endophthalmitis OR scleritis OR albinism OR ((refract*) ADJ3 (error*)) OR ((drooping OR ptotic*) ADJ3 (eyelid*)) OR blepharoptosis OR blepharo-ptosis OR ((midface*) ADJ3 (hypoplas* OR retract*)) OR heterotopia* OR dystopia* OR hyperteloris* OR opthalmopath* OR oculopath* OR glaucom* OR ((eye* OR ocular* OR opthalm* OR lens OR lacrimal* OR strabismus* OR optic* OR pupil* OR retina OR retinal OR sclera OR scleral OR iris OR uvea OR uveal OR orbit* OR conjunctiv* OR accomodat* OR cogan* OR corneal OR fraser OR aicardi OR cone OR Duane-Retraction OR gyrate OR Stargardt OR Walker-Warburg OR Weill-Marchesani OR choroid* OR vitreous) ADJ3 (finding* OR diseas* OR malform* OR disorder* OR abnorm* OR disturb* OR neovascular* OR infect* OR inflammat* OR injur* OR irritat* OR red OR redness OR dry OR

movement* OR pain OR swell* OR toxic* OR hemorrhage* OR fibros* OR prurit* OR tumor* OR tumour* OR myositis OR edema OR syndrom* OR discomfort* OR discharge* OR jaundic* OR fatigue* OR neoplas* OR cancer* OR leukoma OR dystroph* OR cell-loss OR neovasculariz* OR opacit* OR dysplas* OR dystroph* OR atroph* OR hypoplas* OR degenerat* OR ulcer* OR hypertension* OR hypotension*))).ab,ti,kf.) AND (exp Plagiocephaly/ OR (saethre-chotzen OR TCF12 OR heterotop*-ossificat* OR ((apert OR chotzen OR pfeiffer OR craniofrontonas* OR crouzon OR muenke OR ERF OR Carpenter* OR IL11RA) ADJ3 (syndrom* OR diseas*)) OR trigonocephal* OR plagiocephal* OR brachycephal* OR craniostenos* OR craniosynostos* OR ((cranial* OR cranio* OR face* OR facial*) ADJ3 (synostos*))).ab,ti,kf.) AND (exp Child/ OR exp Infant/ OR exp Adolescent/ OR exp "Child Behavior"/ OR exp "Parent Child Relations"/ OR exp "Pediatrics"/ OR "Child Nutrition Sciences"/ OR "Infant nutritional physiological phenomena"/ OR exp "Child Welfare"/ OR "Child Development"/ OR exp "Child Health Services"/ OR exp "Child Care"/ OR "Child Rearing"/ OR exp "Child development Disorders, Pervasive"/ OR "Child Psychiatry"/ OR "Child Psychology"/ OR "Hospitals, Pediatric"/ OR exp "Intensive Care Units, Pediatric"/ OR (adolescen* OR infan* OR newborn* OR (new ADJ born*) OR baby OR babies OR neonat* OR prematur* OR pre-matur* OR child* OR kid OR kids OR toddler* OR teen* OR boy* OR girl* OR minors OR underag* OR (under ADJ1 (age* OR aging OR ageing)) OR juvenil* OR youth* OR kindergar* OR puber* OR pubescen* OR prepubescen* OR prepubert* OR

Cochrane

((vision OR visual OR ptosis OR asthenopia OR eyestrain OR conjunctivit* OR keratitis OR keratoconus OR aniridia OR Anophthalmos OR Blepharophimosis OR Choroidal-Effusion* OR Coloboma OR Ectopia-Lentis OR Familial-Exudative-Vitreoretinopathies OR Hydrophthalmos OR Microphthalmos OR Persistent-Hyperplastic-Primary-Vitreous OR retinitis OR retinoblastoma OR Choroideremia OR Coloboma OR Vitreoretinopath* OR Opthalmopath* OR Leber-Congenital-Amaurosis OR hyphema OR endophthalmitis OR scleritis OR albinism OR ((refract*) NEAR/3 (error*)) OR ((drooping OR ptotic*) NEAR/3 (eyelid*)) OR blepharoptosis OR blepharo-ptosis OR ((midface*) NEAR/3 (hypoplas* OR retract*)) OR heterotopia* OR dystopia* OR hyperteloris* OR opthalmopath* OR oculopath* OR glaucom* OR ((eye* OR ocular* OR opthalm* OR lens OR lacrimal* OR strabismus* OR optic* OR pupil* OR retina OR retinal OR sclera OR scleral OR iris OR uveal OR uvea OR visual* OR orbit* OR conjunctiv* OR accomodat* OR cogan* OR corneal OR fraser OR aicardi OR cone OR Duane-Retraction OR gyrate OR Stargardt OR Walker-Warburg OR Weill-Marchesani OR choroid* OR vitreous) NEAR/3 (finding* OR diseas* OR malform* OR disorder* OR abnorm* OR disturb* OR neovascular* OR infect* OR inflammat* OR injur* OR irritat* OR red OR redness OR dry OR movement* OR pain OR swell* OR toxic* OR hemorrhage* OR fibros* OR prurit* OR tumor* OR tumour* OR myositis OR edema OR syndrom* OR discomfort* OR discharge* OR jaundic* OR fatigue* OR neoplas* OR cancer* OR leukoma OR dystroph* OR cell-loss OR neovasculariz* OR opacit* OR dysplas* OR dystroph* OR atroph* OR hypoplas* OR degenerat* OR ulcer* OR hypertension* OR hypotension*))):ab,ti,kw) AND ((((apert OR chotzen OR pfeiffer OR craniofrontonas* OR crouzon OR muenke OR ERF OR Carpenter* OR IL11RA) NEAR/3 (syndrom* OR diseas*)) OR trigonocephal* OR plagiocephal* OR brachycephal* OR craniostenos* OR craniosynostos* OR ((cranial* OR cranio* OR face* OR facial*) NEAR/3 (synostos*))):ab,ti,kw) AND ((adolescen* OR preadolescen* OR infan* OR newborn* OR (new NEXT/1 born*) OR baby OR babies OR neonat* OR prematur* OR pre-matur* OR child* OR kid OR kids OR toddler* OR teen* OR boy* OR girl* OR minors OR underag* OR (under NEXT/1 (age* OR aging OR ageing)) OR juvenil* OR youth* OR kindergar* OR puber* OR pubescen* OR prepubescen* OR prepubert* OR pediatric* OR paediatric* OR school* OR preschool* OR highschool* OR suckling* OR PICU OR NICU OR PICUs OR NICUs):ab,ti,kw)

Web of Science

TS=(((vision OR visual OR ptosis OR asthenopia OR eyestrain OR conjunctivit* OR keratitis OR keratoconus OR aniridia OR Anophthalmos OR Blepharophimosis OR Choroidal-Effusion* OR Coloboma OR Ectopia-Lentis OR Familial-Exudative-Vitreoretinopathies OR Hydrophthalmos OR

Microphthalmos OR Persistent-Hyperplastic-Primary-Vitreous OR retinitis OR retinoblastoma OR Choroideremia OR Coloboma OR Vitreoretinopath* OR Opthalmopath* OR Leber-Congenital-Amaurosis OR hyphema OR endophthalmitis OR scleritis OR albinism OR ((refract*) NEAR/2 (error*)) OR ((drooping OR ptotic*) NEAR/2 (eyelid*)) OR blepharoptosis OR blepharo-ptosis OR ((midface*) NEAR/2 (hypoplas* OR retract*)) OR heterotopia* OR dystopia* OR hyperteloris* OR opthalmopath* OR oculopath* OR glaucom* OR ((eye* OR ocular* OR opthalm* OR lens OR lacrimal* OR strabismus* OR optic* OR pupil* OR retina OR retinal OR sclera OR scleral OR iris OR uveal OR uvea OR visual* OR orbit* OR conjunctiv* OR accomodat* OR cogan* OR corneal OR fraser OR aicardi OR cone OR Duane-Retraction OR gyrate OR Stargardt OR Walker-Warburg OR Weill-Marchesani OR choroid* OR vitreous) NEAR/2 (finding* OR diseas* OR malform* OR disorder* OR abnorm* OR disturb* OR neovascular* OR infect* OR inflammat* OR injur* OR irritat* OR red OR redness OR dry OR movement* OR pain OR swell* OR toxic* OR hemorrhage* OR fibros* OR prurit* OR tumor* OR tumour* OR myositis OR edema OR syndrom* OR discomfort* OR discharge* OR jaundic* OR fatigue* OR neoplas* OR cancer* OR leukoma OR dystroph* OR cell-loss OR neovasculariz* OR opacit* OR dysplas* OR dystroph* OR atroph* OR hypoplas* OR degenerat* OR ulcer* OR hypertension* OR hypotension*)))) AND (((apert OR chotzen OR pfeiffer OR craniofrontonas* OR crouzon OR muenke OR ERF OR Carpenter* OR IL11RA) NEAR/2 (syndrom* OR diseas*)) OR trigonocephal* OR plagiocephal* OR brachycephal* OR craniostenos* OR craniosynostos* OR ((cranial* OR cranio* OR face* OR facial*) NEAR/2 (synostos*))) AND ((adolescen* OR preadolescen* OR infan* OR newborn* OR (new NEAR/1 born*) OR baby OR babies OR neonat* OR prematur* OR pre-matur* OR child* OR kid OR kids OR toddler* OR teen* OR boy* OR girl* OR minors OR underag* OR (under NEAR/1 (age* OR aging OR ageing)) OR juvenil* OR youth* OR kindergar* OR puber* OR pubescen* OR prepubescen* OR prepubert* OR pediatric* OR paediatric* OR school* OR preschool* OR highschool* OR suckling* OR PICU OR NICU OR PICUs OR NICUs)) NOT ((animal* OR rat OR rats OR mouse OR mice OR murine OR dog OR dogs OR canine OR cat OR cats OR feline OR rabbit OR cow OR cows

Google Scholar

'eye|ocular|optic|ophthalmicdisease|finding|disorder|abnormality'apert|chotzen|pfeiffer|craniofrontonasal|crouzon|muenke syndrome|disease' child|children|pediatric|pediatrics

Supplementary file B. Forest plots and meta-analysis

1.Horizontal strabismus in unicoronal craniosynostosis

Study	Events	Total	Proportion	95%-CI	Weight (common)	Weight (random)
Chung 2015	11	27	0.41	[0.23; 0.60]	13.6%	18.1%
MacIntosh 2007	9	59	0.15	[0.07; 0.26]	29.5%	22.2%
Ntoula 2021	4	16	0.25	[0.06; 0.50]	8.2%	14.7%
Ricci 2007	0	11	0.00	[0.00; 0.15]	5.7%	12.2%
Samra 2015	11	79	0.14	[0.07; 0.23]	39.4%	23.3%
Vasco 2008	3	7	0.43	[0.08; 0.81]	3.7%	9.4%
		**199**				
**Random effects model**			**0.19**	**[0.09; 0.32]**	**--**	**100.0%**

Heterogeneity: $I^2 = 67\%$, $\tau^2 = 0.0173$, $p < 0.01$

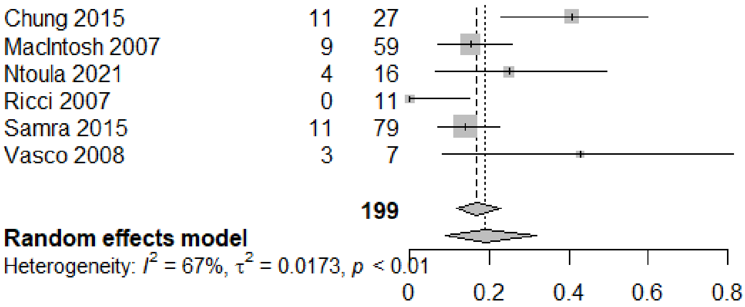

2.Vertical strabismus in unicoronal craniosynostosis

Study	Events	Total	Proportion	95%-CI	Weight (common)	Weight (random)
Chung 2015	1	27	0.04	[0.00; 0.15]	14.6%	21.0%
Gosain 1996	8	14	0.57	[0.30; 0.82]	7.7%	17.4%
MacIntosh 2007	4	59	0.07	[0.01; 0.15]	31.6%	24.0%
Samra 2015	13	79	0.16	[0.09; 0.26]	42.2%	24.7%
Vasco 2008	2	7	0.29	[0.01; 0.68]	4.0%	13.0%
		**186**				
**Random effects model**			**0.17**	**[0.05; 0.33]**	**--**	**100.0%**

Heterogeneity: $I^2 = 80\%$, $\tau^2 = 0.0302$, $p < 0.01$

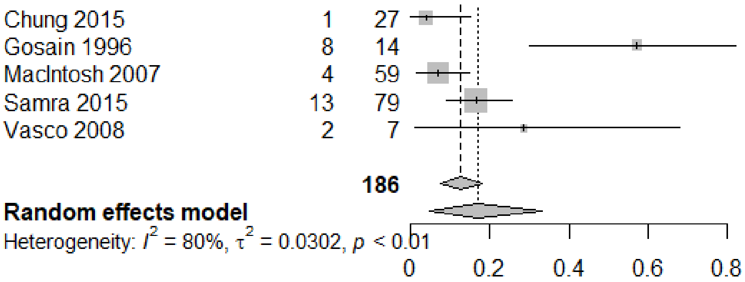

3.Horizontal strabismus in Crouzon syndrome

Study	Events	Total	Proportion	95%-CI	Weight (common)	Weight (random)
Gray 2005	22	71	0.31	[0.21; 0.42]	35.9%	28.2%
Hinds 2021	25	60	0.42	[0.29; 0.54]	30.4%	27.8%
Imai 2013	3	5	0.60	[0.14; 0.98]	2.8%	16.1%
Kreiborg 2010	47	61	0.77	[0.66; 0.87]	30.9%	27.9%
		**197**				
**Random effects model**			**0.52**	**[0.26; 0.76]**	**--**	**100.0%**

Heterogeneity: $I^2 = 91\%$, $\tau^2 = 0.0529$, $p < 0.01$

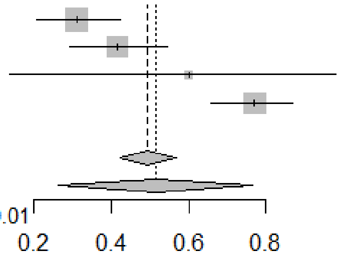

4.Horizontal strabismus in Apert syndrome

Study	Events	Total	Proportion	95%-CI	Weight (common)	Weight (random)
Hinds 2021	29	57	0.51	[0.38; 0.64]	36.2%	36.2%
Imai 2013	2	8	0.25	[0.01; 0.62]	5.3%	5.3%
Khong 2006	34	63	0.54	[0.42; 0.66]	39.9%	39.9%
Khong 2007	13	29	0.45	[0.27; 0.63]	18.6%	18.6%
		**157**				
**Random effects model**			**0.50**	**[0.42; 0.58]**	**--**	**100.0%**

Heterogeneity: $I^2 = 0\%$, $\tau^2 = 0$, $p = 0.47$

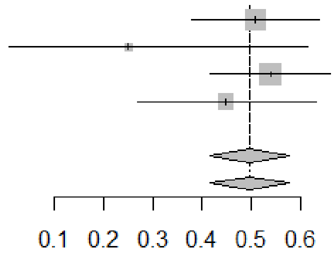

5.Astigmatism in unicoronal craniosynostosis

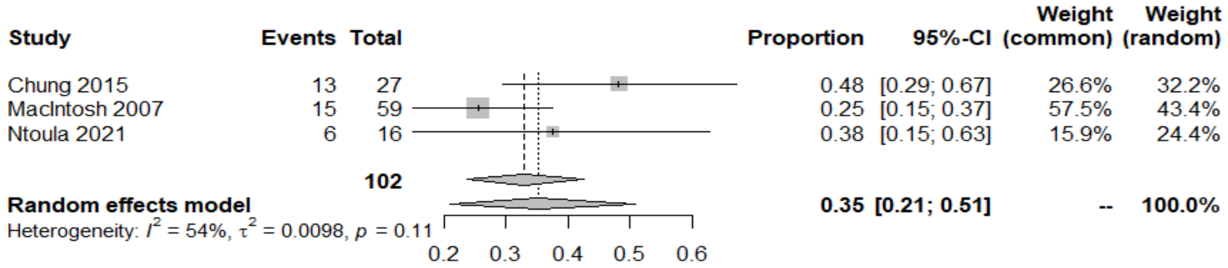

6. Astigmatism in Crouzon syndrome

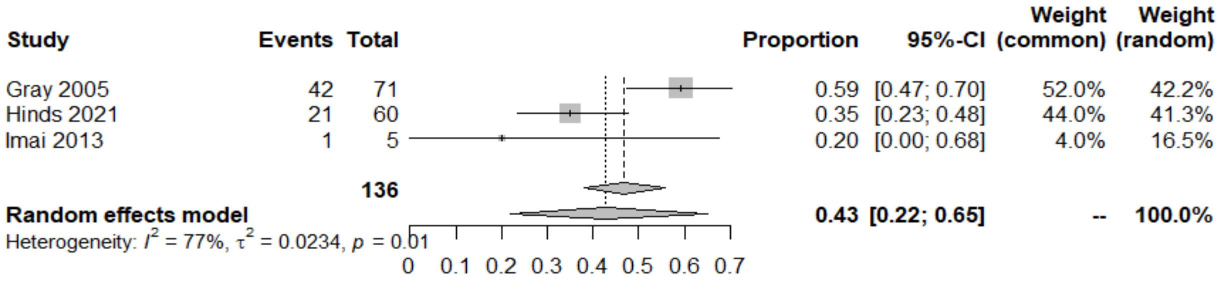

7. Astigmatism in Apert syndrome

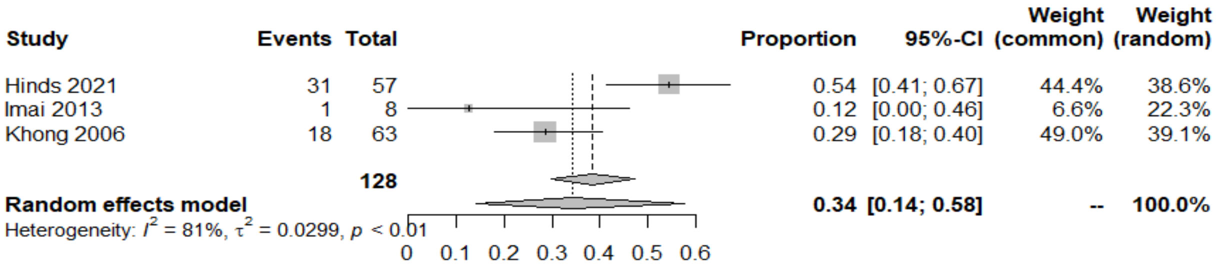

8. Papilledema in non-syndromal craniosynostosis

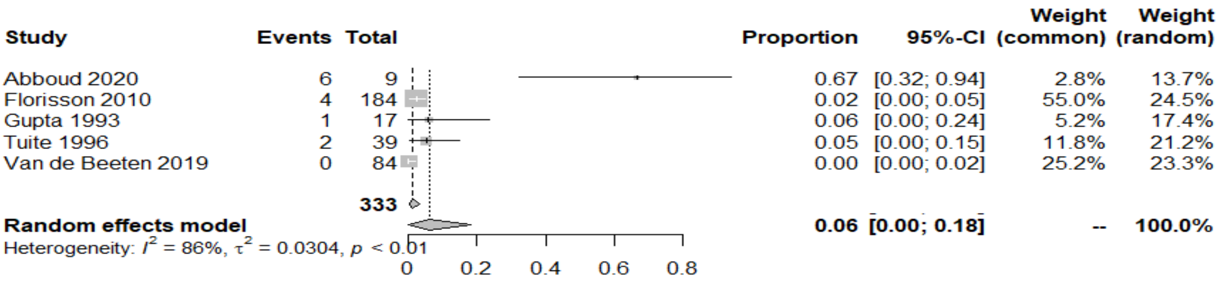

9. Papilledema in non-syndromal craniosynostosis (without study of Abboud et al.2020)

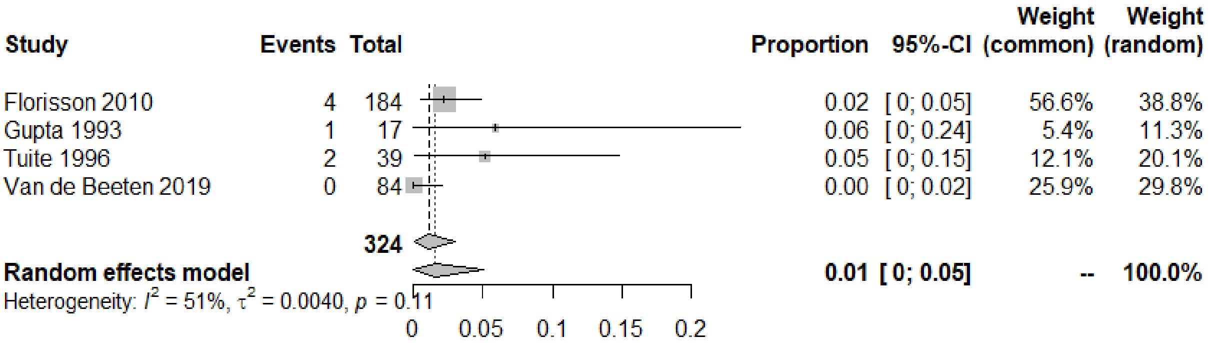

10. Papilledema in syndromale craniosynostosis

Study	Events	Total	Proportion	95%-CI	Weight (common)	Weight (random)
Bannink 2008	25	66	0.38	[0.26; 0.50]	13.8%	10.9%
Gray 2005	11	71	0.15	[0.08; 0.25]	14.9%	11.0%
Gupta 1993	2	5	0.40	[0.02; 0.86]	1.1%	3.2%
Jadico 2006	3	21	0.14	[0.02; 0.33]	4.5%	7.5%
Jong, de 2010	32	121	0.26	[0.19; 0.35]	25.3%	12.1%
Kim 2019	2	15	0.13	[0.00; 0.36]	3.2%	6.3%
Liasis 2006	1	8	0.12	[0.00; 0.46]	1.8%	4.4%
Ottenlander, den 2019	3	38	0.08	[0.01; 0.19]	8.0%	9.4%
Sharma 2016	1	22	0.05	[0.00; 0.18]	4.7%	7.6%
Spruijt 2016	13	37	0.35	[0.20; 0.51]	7.8%	9.3%
Stavrou 1997	2	4	0.50	[0.03; 0.97]	0.9%	2.7%
Tuite 1996	11	55	0.20	[0.10; 0.32]	11.6%	10.4%
Van de Beeten 2019	0	11	0.00	[0.00; 0.15]	2.4%	5.3%
		**474**				
**Random effects model**			**0.18**	**[0.11; 0.26]**	**--**	**100.0%**

Heterogeneity: $I^2 = 65\%$, $\tau^2 = 0.0134$, $p < 0.01$

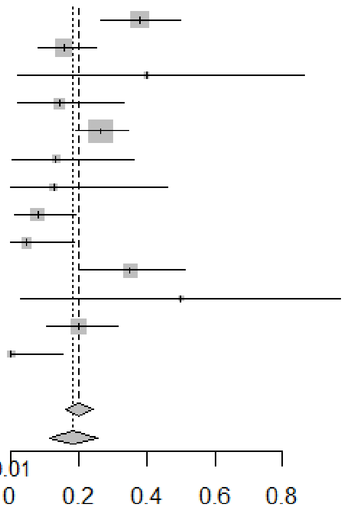

11. Papilledema in Crouzon syndrome

Study	Events	Total	Proportion	95%-CI	Weight (common)	Weight (random)
Gray 2005	11	71	0.15	[0.08; 0.25]	39.6%	18.9%
Gupta 1993	1	4	0.25	[0.00; 0.79]	2.5%	8.6%
Jong, de 2010	24	45	0.53	[0.39; 0.68]	25.2%	18.1%
Kim 2019	2	15	0.13	[0.00; 0.36]	8.6%	14.7%
Spruijt 2016	11	19	0.58	[0.35; 0.79]	10.8%	15.6%
Stavrou 1997	2	4	0.50	[0.03; 0.97]	2.5%	8.6%
Tuite 1996	7	19	0.37	[0.16; 0.60]	10.8%	15.6%
		**177**				
**Random effects model**			**0.34**	**[0.17; 0.53]**	**--**	**100.0%**

Heterogeneity: $I^2 = 78\%$, $\tau^2 = 0.0397$, $p < 0.01$

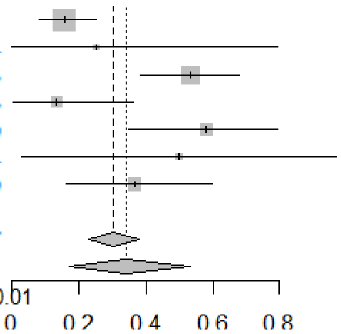

12. Papilledema in Apert syndrome

Study	Events	Total	Proportion	95%-CI	Weight (common)	Weight (random)
Gupta 1993	1	1	1.00	[0.00; 1.00]	2.4%	2.8%
Jong, de 2010	2	22	0.09	[0.00; 0.25]	36.6%	34.7%
Liasis 2006	1	8	0.12	[0.00; 0.46]	13.8%	14.9%
Spruijt 2016	2	18	0.11	[0.00; 0.31]	30.1%	29.5%
Tuite 1996	2	10	0.20	[0.01; 0.51]	17.1%	18.0%
		**59**				
**Random effects model**			**0.09**	**[0.01; 0.22]**	**--**	**100.0%**

Heterogeneity: $I^2 = 11\%$, $\tau^2 = 0.0026$, $p = 0.35$

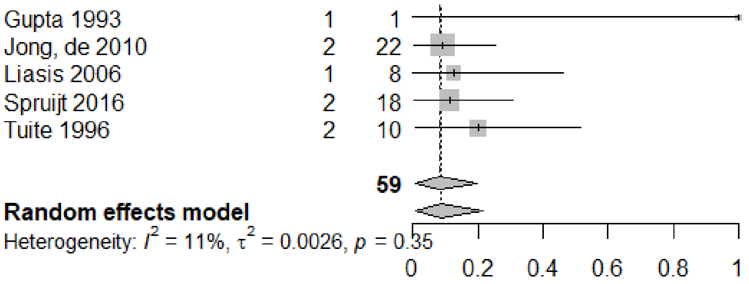

13. Papilledema in Apert syndrome (without Gupta et al., 1993)

Study	Events	Total	Proportion	95%-CI	Weight (common)	Weight (random)
Jong, de 2010	2	22	0.09	[0.00; 0.25]	37.5%	37.5%
Liasis 2006	1	8	0.12	[0.00; 0.46]	14.2%	14.2%
Spruijt 2016	2	18	0.11	[0.00; 0.31]	30.8%	30.8%
Tuite 1996	2	10	0.20	[0.01; 0.51]	17.5%	17.5%
		**58**				
**Random effects model**			**0.11**	**[0.04; 0.22]**	**--**	**100.0%**

Heterogeneity: $I^2 = 0\%$, $\tau^2 = 0$, $p = 0.85$

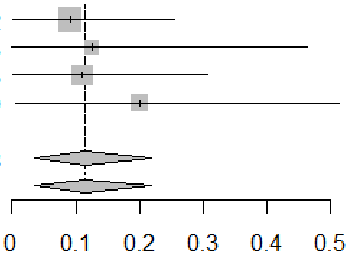

14. Proptosis in syndromal craniosynostosis

Study	Events	Total	Proportion	95%-CI	Weight (common)	Weight (random)
Albuquerque 2004	5	5	1.00	[0.68; 1.00]	3.2%	16.8%
Jadico 2006	3	21	0.14	[0.02; 0.33]	12.3%	20.3%
Khong 2006	55	63	0.87	[0.78; 0.95]	36.4%	21.3%
Kreiborg 2010	61	61	1.00	[0.97; 1.00]	35.2%	21.3%
Sharma	21	22	0.95	[0.82; 1.00]	12.9%	20.4%
		**172**				
**Random effects model**			**0.86**	**[0.52; 1.00]**	**--**	**100.0%**

Heterogeneity: $I^2 = 95\%$, $\tau^2 = 0.1494$, $p < 0.01$

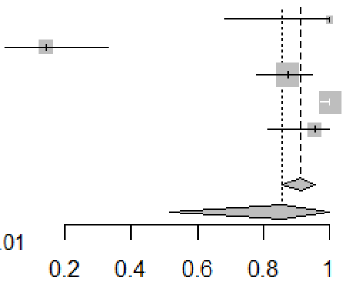

15. Proptosis in syndromal craniosynostosis (Without Jadico et al., 2006)

Study	Events	Total	Proportion	95%-CI	Weight (common)	Weight (random)
Albuquerque 2004	5	5	1.00	[0.68; 1.00]	3.6%	12.4%
Khong 2006	55	63	0.87	[0.78; 0.95]	41.5%	31.5%
Kreiborg 2010	61	61	1.00	[0.97; 1.00]	40.2%	31.3%
Sharma	21	22	0.95	[0.82; 1.00]	14.7%	24.8%
		**151**				
**Random effects model**			**0.98**	**[0.87; 1.00]**	**--**	**100.0%**

Heterogeneity: $I^2 = 75\%$, $\tau^2 = 0.0230$, $p < 0.01$

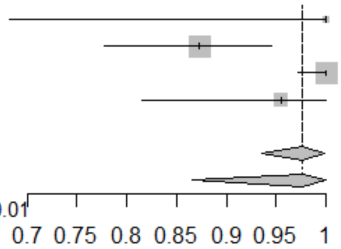

16. Esotropia in unicoronal craniosynostosis

Study	Events	Total	Proportion	95%-CI	Weight (common)	Weight (random)
Chung 2015	5	27	0.19	[0.06; 0.36]	22.4%	24.0%
MacIntosh 2007	5	59	0.08	[0.02; 0.17]	48.6%	31.3%
Ntoula 2021	1	16	0.06	[0.00; 0.25]	13.5%	18.6%
Ricci 2007	0	11	0.00	[0.00; 0.15]	9.4%	15.0%
Vasco 2008	3	7	0.43	[0.08; 0.81]	6.1%	11.1%
		**120**				
**Random effects model**			**0.10**	**[0.02; 0.21]**	**--**	**100.0%**

Heterogeneity: $I^2 = 50\%$, $\tau^2 = 0.0119$, $p = 0.09$

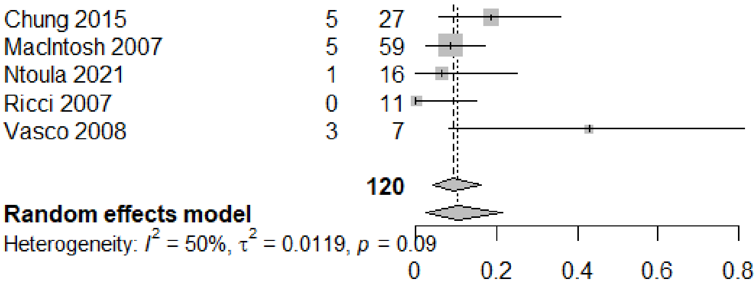

17. Esotropia in unicoronal craniosynostosis (Without Vasco et al., 2008)

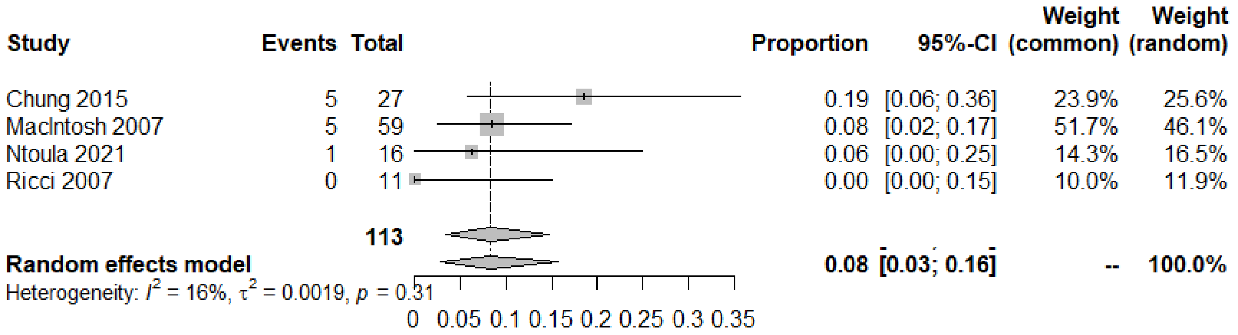

18. Exotropia in unicoronal craniosynostosis

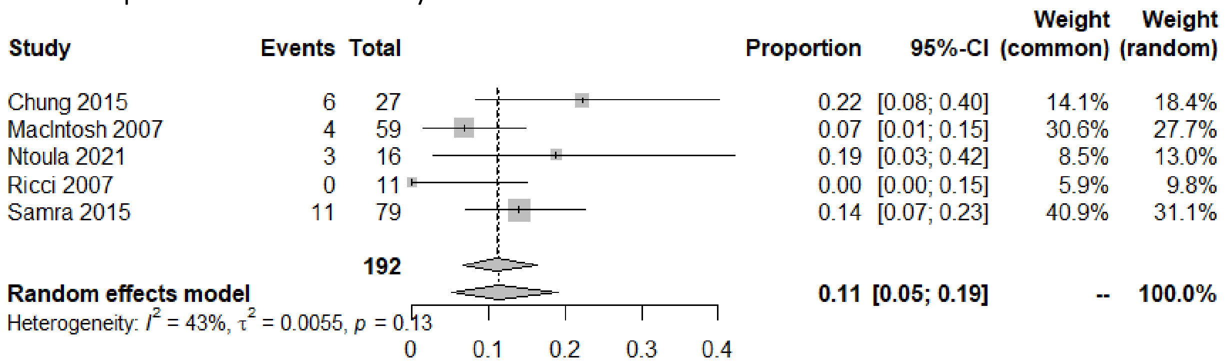

19. Anisometropia in unicoronal craniosynostosis

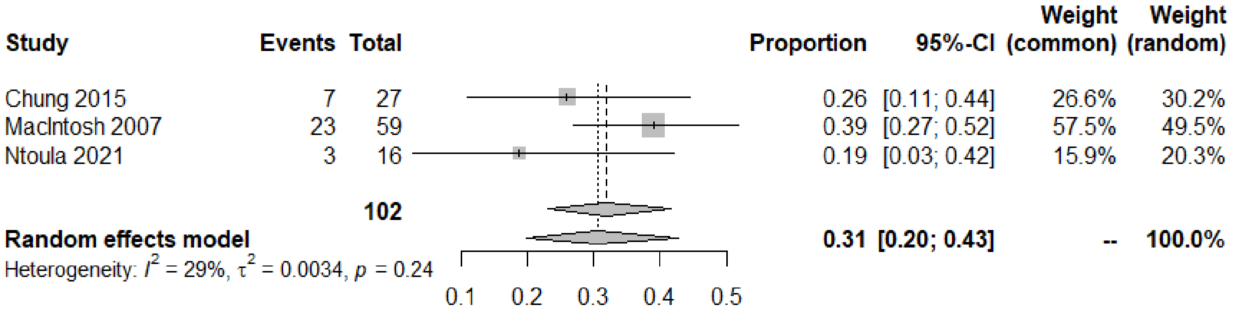

20. Strabismus (both horizontal and vertical) in non-syndromal craniosynostosis

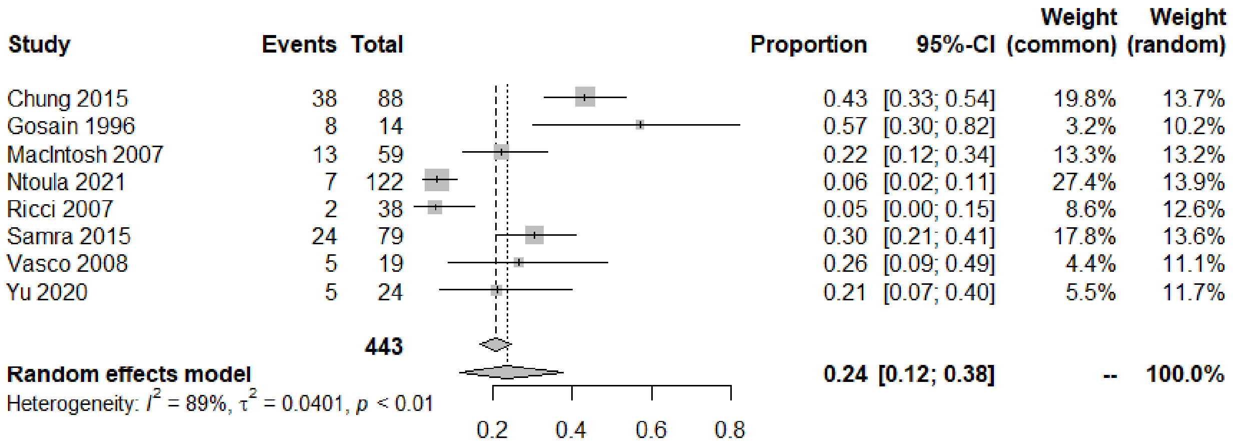

21. Strabismus (both horizontal and vertical) in syndromal craniosynostosis

Study	Events	Total	Proportion	95%-CI	Weight (common)	Weight (random)
Gray 2005	24	71	0.34	[0.23; 0.45]	15.9%	13.5%
Hinds 2021	72	165	0.44	[0.36; 0.51]	36.9%	14.1%
Imai 2013	5	13	0.38	[0.13; 0.67]	3.0%	10.3%
Jadico 2006	21	21	1.00	[0.92; 1.00]	4.8%	11.6%
Khong 2006	37	63	0.59	[0.46; 0.71]	14.1%	13.4%
Khong 2007	13	29	0.45	[0.27; 0.63]	6.6%	12.2%
Kreiborg 2010	47	61	0.77	[0.66; 0.87]	13.7%	13.3%
Sharma 2016	10	22	0.45	[0.25; 0.67]	5.0%	11.7%
		**445**				
**Random effects model**			**0.58**	**[0.41; 0.73]**	**--**	**100.0%**

Heterogeneity: $I^2 = 90\%$, $\tau^2 = 0.0451$, $p < 0.01$

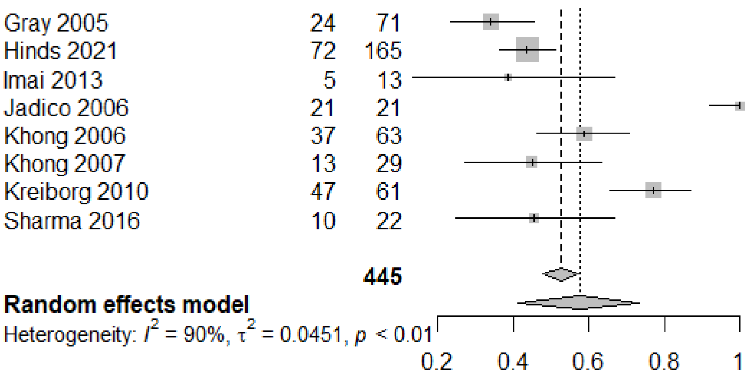

22. Exotropia in Crouzon syndrome

Study	Events	Total	Proportion	95%-CI	Weight (common)	Weight (random)
Gray 2005	16	71	0.23	[0.13; 0.33]	35.9%	27.4%
Hinds 2021	21	60	0.35	[0.23; 0.48]	30.4%	27.2%
Imai 2013	3	5	0.60	[0.14; 0.98]	2.8%	18.1%
Kreiborg 2010	47	61	0.77	[0.66; 0.87]	30.9%	27.2%
		**197**				
**Random effects model**			**0.47**	**[0.18; 0.77]**	**--**	**100.0%**

Heterogeneity: $I^2 = 93\%$, $\tau^2 = 0.0785$, $p < 0.01$

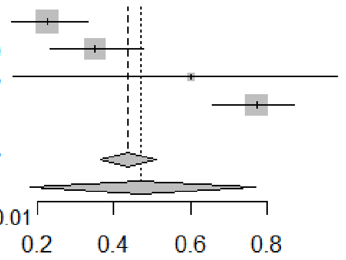

23. Exotropia in Apert syndrome

Study	Events	Total	Proportion	95%-CI	Weight (common)	Weight (random)
Hinds 2021	27	57	0.47	[0.34; 0.60]	44.4%	43.6%
Imai 2013	2	8	0.25	[0.01; 0.62]	6.6%	10.2%
Khong 2006	21	63	0.33	[0.22; 0.46]	49.0%	46.1%
		**128**				
**Random effects model**			**0.38**	**[0.27; 0.50]**	**--**	**100.0%**

Heterogeneity: $I^2 = 33\%$, $\tau^2 = 0.0033$, $p = 0.23$

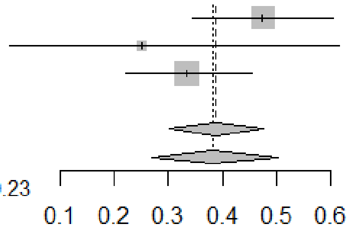

Supplement: Supplementary file 1 [file jcm-11-01060-s001.zip › jcm-1571368-supplementary.pdf]
